# Preoperative Anxiety among Adult Patients Undergoing Elective Surgeries at a Tertiary Teaching Hospital: A Cross-Sectional Study during the Era of COVID-19 Vaccination

**DOI:** 10.3390/healthcare10030515

**Published:** 2022-03-11

**Authors:** Abdelkarim Aloweidi, Sami Abu-Halaweh, Mahmoud Almustafa, Zaineh Marei, Sara Yaghi, Lina Hababeh, Neebal Al-Gallab, Shatha Al-Jaberi, Lina Ghattas, Sham Romeo Alrabadi, Anas Al-Oweidi, Isam Bsisu

**Affiliations:** 1 Department of Anesthesia and Intensive Care, School of Medicine, The University of Jordan, Amman 11942, Jordan; akaloweidi@hotmail.com (A.A.); s.halaweh@ju.edu.jo (S.A.-H.); m.al-mustafa@ju.edu.jo (M.A.); 2 Department of Undergraduate Studies, School of Medicine, The University of Jordan, Amman 11942, Jordan; z.s.mari98@gmail.com (Z.M.); sarayaghi0@gmail.com (S.Y.); linahababeh@gmail.com (L.H.); nebal_algallab@yahoo.com (N.A.-G.); shathajaberi@yahoo.com (S.A.-J.); linaghattas199@gmail.com (L.G.); sdoctors1998@gmail.com (S.R.A.); anasaloweidi@gmail.com (A.A.-O.)

**Keywords:** anxiety, perioperative medicine, COVID-19

## Abstract

Anxiety in the perioperative period has significant impact on both the flow of surgery and the post-operative recovery process. The aim of this cross-sectional study is to determine the prevalence of preoperative anxiety among adult patients undergoing elective surgical procedures at a tertiary teaching hospital and the effect of COVID-19 and COVID-19 vaccines on preoperative anxiety. We used the Amsterdam Preoperative Anxiety and Information Scale (APAIS) to assess patients’ anxiety toward surgery and their need for more information. Patients with APAIS about anesthesia and surgery (APAIS-A-T) total score <10 were considered as the low preoperative anxiety group, while patients with APAIS-A-T ≥11 were considered as the high preoperative anxiety group. The overall APAIS-A-T score of the 794 included patients was 8.5 ± 4.5. The mean APAIS-A-T score was 7.0 ± 3.8 among males and 9.6 ± 4.6 among female patients (*p* < 0.001). The APAIS-A-T score for those who had previously underwent surgery under anesthesia was 8.3 ± 4.4, compared to 9.5 ± 4.8 for those who had not (*p* = 0.002). No significant difference was found between those with a previous history of COVID-19 and those without (*p* = 0.105), nor between those who were vaccinated and those who were not (*p* = 0.550). Sixty-four (26.8%) highly anxious patients were afraid of becoming infected with COVID-19 during their hospital stay (*p* = 0.009). This fear of COVID-19 in-hospital transmission made 19 (7.9%) highly anxious patients and 36 (4.5%) of the total sample hesitant to undergo this surgery (*p* = 0.002). In conclusion, this study demonstrated that 30.1% of patients had high preoperative anxiety, with fear of pain after surgery being the most common factor related to anxiety on the day of surgery. Controlling the spread of COVID-19 can play a crucial role in decreasing preoperative anxiety during this pandemic.

## 1. Introduction

A staggering 243.2 million surgical operations are performed worldwide each year, of which 7 million patients suffer a major complication and 1 million succumb to death [1]. These concerning statistics emphasize the need to become more vigilant and focus on the operative field in order to improve the healthcare outcome. It is without a doubt that the possibility of complications within surgery imparts stress to the patient and ultimately entails a traumatic situation [2]. Preoperative anxiety, feelings of discomfort and tension, along with other physical and autonomic symptoms, therefore results before surgery [3,4]. A 2020 study found that 48% of surgical patients are anxious before their surgery [5]. This anxiety will have an impact on both the flow of the surgery and the post-operative recovery process [6,7,8].

The COVID-19 pandemic, its continuously escalating numbers of confirmed cases, and the development of research regarding its complications have consequently led to an increase in public awareness and concern regarding the severity of contracting the virus [9]. A myriad of studies has revealed a correlation between this pandemic and anxiety [10]. Since hospitals are a potential source of infection, visiting a hospital, especially if you are an in-patient, will elevate your level of anxiety, especially in light of the pandemic. Despite the increasing public acceptance and interest toward vaccinations, anxiety and fear are spreading among populations due to both reports on vaccine side effects and the spread of pseudoscientific rumors [11,12]. Preoperative anxiety is worsened by these factors in combination, thus necessitating an intervention to prevent it.

According to previous studies, preoperative anxiety can be explained by concern over anesthesia error, fatigue, drowsiness, and fear of complications and death [13,14]. Fear of becoming infected with COVID-19 during hospitalization has become a major contributing factor to perioperative anxiety [15].

As there is still a paucity of information concerning this topic, the main objective of our study is therefore to determine the prevalence of preoperative anxiety among surgical patients at our tertiary teaching hospital and whether the COVID-19 pandemic aggravates the problem. Moreover, we will determine whether vaccination against COVID-19 would affect preoperative anxiety, and we will evaluate the factors associated with preoperative anxiety within our study population.

## 2. Materials and Methods

### 2.1. Design

This cross-sectional observational study was conducted at Jordan University Hospital (JUH), which is a tertiary teaching hospital located in Amman, the capital of the Hashemite Kingdom of Jordan. Data collection was carried out over a period of three months, between 19 September and 19 December 2021.

### 2.2. Inclusion and Exclusion Criteria

The targeted population was adult patients who are 18 years and older, from both genders, who were planned for elective surgical procedures under general anesthesia, neuraxial anesthesia, or regional anesthesia during the study timeframe, whether they had been previously infected with COVID-19 or not.

We excluded the following patient groups from our study: any patient aged less than 18 years at the time of their surgery, patients previously diagnosed with psychiatric illnesses, patients who refused to participate and who withdrew from the investigation, patients who have previously participated in this study and are undergoing another surgery, and patients undergoing emergency operations.

### 2.3. Ethical Approval

A written informed consent was obtained from all participants prior to their voluntary enrollment. Data collection did not include any identifying questions, and data were handled with the utmost confidentiality. Entries were coded via patients’ file numbers as identifiers and were used solely for statistical analysis. An approval from the Institutional Review Board (IRB) committee of JUH was obtained (number: 10/2022/2441).

### 2.4. Questionnaire

The questionnaire was filled via face-to-face interviews, in which patients were questioned by 6th year medical students upon their arrival to the preoperative holding area one hour prior to their surgical procedure. The questionnaire was divided into 4 sections. The first section included sociodemographic information, including age, gender, level of education, whether they resided in urban or rural area, their average income, and their occupation. The second section investigated patients’ personal and family medical and surgical history, if they had history of COVID-19 infection, vaccination history, and previous complications due to COVID-19. In addition, we inquired about the number of times they had previously undergone anesthetic or surgical procedures, and if they had any complications related to those procedures. The third section included factors precipitating preoperative anxiety. The fourth section included the Amsterdam Preoperative Anxiety and Information Scale (APAIS) [16].

APAIS is defined as a self-report questionnaire that aims to assess patients’ anxiety toward surgery and their need for more information. It has six questions that evaluate three separate areas: anxiety about anesthesia, anxiety about surgery and the need for information. The items are rated on a five-point Likert scale, with (1): “not at all” and (5): “extremely” (Supplementary Table S1). The highest scores of the two items related to anxiety about anesthesia (APAIS-A-An) and surgery (APAIS-A-Su) were 10 each, resulting in a maximum total score of 20 for total preoperative anxiety (APAIS-A-T). The maximum score for the items investigating patients’ need for information (APAIS-I) was 10. Patients who scored ≥11 on APAIS-A-T score were considered to have high preoperative anxiety. The scale was translated to the Arabic language, and the translation was reviewed by the investigators to ensure preservation of identical meaning of all of its items. All interviews were carried out in the Arabic language, which is the official language of Jordan.

### 2.5. Statistical Analysis

We used Statistical Package for Social Science program (SPSS) version 23.0 (IBM, Armonk, NY, USA) to perform statistical analyses. After performing the descriptive analysis, Kolmogorov–Smirnov test, skewness, and kurtosis were used to test for central tendency and normality of distribution. We compared between patients with high and low preoperative anxiety using Pearson’s Chi-squared (χ2) test for categorical variables and Mann–Whitney U test for ordinal or continuous variables. Comparisons between different surgical specialties in APAIS scores were performed using Kruskal–Wallis one-way analysis of variance. Factors associated with preoperative anxiety, history of COVID-19, and history of COVID-19 vaccination were compared between patients with high and low preoperative anxiety using χ2 test. Univariable binary logistic regression analysis was applied to predict factors associated with high preoperative anxiety. Odds ratio (OR) and the 95% confidence interval (95% CI) of OR were illustrated. Variables that were significant in the univariable binary regression model were included in a multivariable binary logistic regression analysis. A *p* value < 0.05 was used as a threshold for statistical significance.

## 3. Results

This study included a total of 794 patients with a mean age of 44.9 ± 16.1 years, of which 479 (60.3%) were female, while 315 (39.7%) were male. The overall APAIS-A-T score of the studied population was 8.5 ± 4.5. We used the aforementioned score to divide patients into two groups, high preoperative anxiety group (group B), which included 239 (30.1%) patients, and low preoperative anxiety group (group A), which included 555 (69.9%) patients, as presented in Table 1. Upon comparing between the two groups, we found significant difference in their age (*p* < 0.001), where the mean age of highly anxious patients was 41.2 ± 15.4 years, which is less than that of group A (46.5 ± 16.1 years). In addition, 179 out of 479 females were highly anxious, marking an anxiety rate of 37.4% among females, compared to 19% (60 out of 315) among males (*p* < 0.001). The mean APAIS-A-T score was 7.0 ± 3.8 among males, while it was 9.6 ± 4.6 among female patients (*p* < 0.001). No significant differences were found between the two groups in relation to other included demographic factors (Table 1).

We found a significant difference between the two groups in their previous operative history, with 443 (79.8%) of group A having a previous history of surgery under anesthesia (*p* = 0.005). The APAIS-A-T score for those who had previously undergone surgery under anesthesia was 8.3 ± 4.4, compared to 9.5 ± 4.8 for those who had not (*p* = 0.002). In comparison, previous history of anesthetic or surgical complications among those with previous surgeries was not found to be significantly different between the two groups (*p* = 0.067 and 0.208, respectively). Upon comparing between different surgical specialties in the mean APAIS-A-T score (*p* < 0.001), we found that the higher scores were among oncological surgeries (11.5 ± 5.2), followed by gynecological and obstetric surgeries (9.4 ± 4.7) and cardiac and vascular surgeries (9.2 ± 4.9) (Table 2).

Factors making patients anxious on the day of surgery are illustrated in Table 3. Notably, fear of pain after surgery was the most common factor among the studied population (*n* = 274; 34.5%), which was significantly higher among highly anxious patients (*n* = 118; 49.4%; *p* < 0.001).

Among the included sample, 229 (28.8%) had previous history of being diagnosed with COVID-19 infection, while 653 of the studied sample had received COVID-19 vaccination, marking a vaccination rate of 82.2% among our adult operative population at the time of this investigation. Forty-one (17.9%) of the 229 patients with previous history of COVID-19 were afraid of becoming infected with COVID-19 during their hospital stay (*p* = 0.168), while 134 (20.5%) of the 653 vaccinated individuals were afraid of becoming infected during their hospital stay (*p* = 0.446). When comparing the mean APAIS-A-T score, no significant difference was found between those with previous history of COVID-19 infection and those without (*p* = 0.105), nor between those who were vaccinated and those who were not (*p* = 0.550). Eighty-three (34.7%) of those who had high anxiety had previous history of having family members or friends who experienced previous complications of COVID-19 (*p* = 0.002), 64 (26.8%) of those highly anxious patients were afraid of becoming infected with COVID-19 during their hospital stay (*p* = 0.009). This fear of COVID-19 in-hospital transmission made 19 (7.9%) highly anxious patients and 36 (4.5%) of the total sample hesitant to undergo surgery (*p* = 0.002) (Table 4).

We performed univariable binary regression analysis to predict factors related to high preoperative anxiety and included significant correlations in the multivariable binary regression model (Table 5). We found that age (OR: 0.98; 95% CI: 0.97–0.99; *p* < 0.001), male gender (OR: 0.39; 95% CI: 0.28–0.56; *p* < 0.001), and previous history of surgery under anesthesia (OR: 0.63; 95% CI: 0.43–0.91; *p* = 0.014) were negatively associated with high anxiety. In comparison, being afraid of contracting COVID-19 during hospital stay was positively associated with high anxiety (OR: 1.47; 95% CI: 1.02–2.14; *p* = 0.041).

## 4. Discussion

New experiences often provoke anxiety. One of those experiences could be surgery; the preoperative period is the most stressful factor for the patient. If this fact is left unrecognized, it will negatively affect the recovery period and control of post-operative pain [17]. Despite the extensive research on preoperative anxiety and its varied causes, there are few studies examining the impact of the COVID-19 pandemic on anxiety before surgery, especially in our region. This is the first study to be conducted in the Jordanian teaching hospital regarding this topic. This investigation includes all surgical specialties, taking into consideration different age groups and psychosocial factors. Furthermore, it also addresses how COVID-19 infection and vaccination may affect preoperative anxiety.

### 4.1. Prevalence of Preoperative Anxiety 

Of our 794 participants, 60.3% were female and 39.7% were male. The study found that the average total APAIS anxiety score (APAIS-A-T) was 8.5 ± 4.5. In total, 30.1% of respondents reported high anxiety (defined as APAIS-A-T > 10), which is consistent with a German study in which mean anxiety scores were reported to be 9.9%, and 40% were considered highly anxious [3]. However, studies conducted in Ethiopia, Nepal and Sri Lanka found the prevalence to be 61%, 51.8% and 76.7%, respectively [14,18,19]. Different scoring systems, different sample sizes, higher female involvement, and the selection of surgical specialties may explain this discrepancy.

### 4.2. Factors Related to Preoperative Anxiety

Preoperative anxiety encompasses both the fear of surgery and anesthesia. However, few studies have addressed whether patients are more anxious about surgery or anesthesia. According to several studies [3,20], the average level of anxiety related to surgery was higher. Our data support this since the mean anxiety score for the surgery was 4.6 ± 2.8, while it was 3.9 ± 2.5 for the anesthesia.

Results of our study suggest that 179 out of 479 females were highly anxious, marking an anxiety rate of 37.4% among females, compared to 19% among males (*p* < 0.001). This goes in line with the findings of a prior German study, which found that females are more likely to suffer from preoperative anxiety [13]. A similar conclusion was reached by two studies investigating preoperative anxiety in Ethiopian [14] and Turkish [21] populations. In the current study, the mean APAIS-A-T score was higher among gynecological and obstetric surgeries (9.4 ± 4.7), when compared to urological surgeries (7.1 ± 3.9). Even within urological procedures (*n* = 146), when we compared between males (*n* = 111; 76%) and females (*n* = 35; 24%), the mean APAIS-A-T score was 6.4 ± 3.6 for males, compared to 9.2 ± 4.4 for females (*p* < 0.001). Women in Jordan, which is a Middle Eastern country, tend to dress modestly due to cultural and religious reasons. During surgery, they must wear surgical gowns instead of their traditional clothes, which could make them uncomfortable, hence increasing their anxiety. Therefore, we hypothesize that this may be one of the reasons why our female population had higher anxiety scores compared to the studies mentioned above. Moreover, the gender of the anesthesiologist and the surgeon must be taken into consideration in future studies in our region. In our center, almost all of the urologists are males, while in the obstetrics and gynecology department and anesthesia department, there are nearly equal numbers of healthcare providers from both genders.

In addition, our results confirm findings of previous studies that found that previous anesthetic experiences were significantly associated with preoperative anxiety [13]. An additional factor of preoperative anxiety is related to the surgical discipline; our study compares average APAIS-A-T score between different surgical disciplines. Surgery in the field of oncology had the highest score (11.5 ± 5.2), followed by gynecological, obstetric surgery (9.4 ± 4.7) and cardiac and vascular surgery (9.2 ± 4.9). This verifies an Italian study’s conclusion that oncological patients experience a higher value of state anxiety [15]. Oncology by its nature is frightening to human beings, as it is associated with the notion of death. Our oncological patient sample was small; future studies are required in oncological centers to further compare and investigate whether factors within oncological patients themselves affect their state of anxiety, and to what extent.

Preoperative anxiety is negatively correlated with age. Therefore, participants below the average age were more anxious compared to older participants in our study. This finding ties well with studies conducted in Turkey and Ethiopia [21,22].

Another factor that is addressed in prior studies is the patient’s educational level. The more educated they are, the more likely they are to be cognizant of procedural details, thus decreasing preoperative anxiety, but we found this factor to be non-significant in our study [23].

### 4.3. The Reason for Preoperative Anxiety

The worry that patients experience awaiting their surgeries can be explained by a variety of factors. Our study highlighted the most significant factors contributing to preoperative anxiety by suggesting multiple probable fears that might be felt, including surgical complications, anesthesia, postoperative pain, doctor’s mistakes, death and others. Notably, fear of pain after surgery was the most common factor among the studied population (34.5%). This result was in line with a similar prospective cohort study performed at Ahi Evran University [24]. The highest percentage obtained from the methods in the literature regarding fear of pain to be the most concerning factor was 22%, by the aforementioned study; however, our study resulted in 34.5%.

Concern that was not explained by any of the suggested factors (33.1%) and fear of surgical complications (31.7%) took second and third places, respectively, in the order of most concerning factors preceded only by fear of pain, contrary to the results provided by a cross-sectional study [14] where concern about surgical complications ranked the first and fear of pain ranked the third among the most worrying factors. The reason behind this inconsistency in results might be attributed to the assumption that patients in our study were not thoroughly counseled about post-operative pain management strategies, unlike other centers where counseling services were more successful in reducing fear of postoperative pain.

A cross-sectional study involving 239 patients [25] found that fear of death was the most concerning factor preoperatively, which was inconsistent with our study where fear of death was the least concerning of the mentioned factors. A plausible explanation could be the differences in cultures and beliefs between the two populations.

Our findings reveal that fear of doctor’s mistakes is ranked fourth, fear of anesthesia fifth and fear of becoming infected with COVID-19 sixth of the most common patient fears. The percentages of patients whose greatest fears were those factors are 27.8%, 25.3% and 12.2%, respectively.

### 4.4. Preoperative Anxiety and COVID-19

Within the study population, 28.8% were previously infected with COVID-19, and 82.2% had taken at least one dose of the COVID-19 vaccine, while of the Jordanian population as a whole, 10% had already been infected and 41.6% had taken a COVID-19 vaccine at the time this study was conducted, according to the WHO [26]. This statistical difference is owed to the inclusion of older adults who suffer from comorbid conditions, which makes them more vulnerable to infection. As for the vaccination, the reason for this difference was due to the exclusion of participants below the age of 18 in our study, because COVID-19 vaccines only started being administered to them in July 2021 [27].

The findings of our study reveal that 21% of patients felt anxious regarding contracting COVID-19. Comparatively, a study conducted by Doglietto and colleagues in eastern Lombardy, Italy demonstrated that figure to be 30.3% [15]. In order to fully appreciate the difference in our statistical findings, it is imperative to note that eastern Lombardy was severely impacted by the COVID-19 outbreak and that the study was performed instantly after lockdown. Our study, however, was conducted when coronavirus constraints were being markedly reduced and when public knowledge about COVID-19 was increasing. We consequently drew different results due to the differing contexts of our studies.

Our main finding was that patients classified as highly anxious were more afraid of becoming infected with COVID-19 during their hospital stay than those with low anxiety, with the percentages of those afraid being 26.8% and 18.6%, respectively. These results are in line with the findings of a study carried out in Pakistan. That study reported that 74.5% of people avoided or reduced their visits to healthcare facilities during the pandemic [28]. Anxiety among pregnant woman in follow-up visits during the pandemic were also reported in another study [29]. Fear of contracting COVID-19 during hospital transmission made 7.9% of highly anxious patients and 4.5% of the total sample hesitant to undergo surgery. Furthermore, a significant correlation was established between highly anxious patients and having family members and/or friends who experienced previous COVID-19 related complications. This correlation was also highlighted in a previous Iranian study, which revealed that anxiety was significantly higher among people whose family members, relatives, or friends had contracted COVID-19 [30].

A key finding that emerged was that preoperative anxiety related to COVID-19 infection did not significantly differ between those who had previously contracted coronavirus and those who had not. This can be attributable to the fact that 57.5% of those who had been previously infected experienced only mild symptoms, and 10.9% experienced no symptoms. Furthermore, the intensity of anxiety fluctuated during the pandemic and currently, since almost all the country returned to pre-COVID-19 normal life, the impact of COVID-19 on preoperative anxiety has become less. In addition, the results show that vaccination does not seem to significantly impact a patient’s preoperative anxiety. This can be elucidated by the spread of social misinformed rumors surrounding the effectiveness of vaccines, as well as the lack of confidence in vaccine safety [11]; therefore, these factors prevented almost half of our study population from feeling safe after taking the vaccine.

### 4.5. Limitations

This study had several limitations. First, this study was a single-center study, which enabled us to evaluate preoperative anxiety at a tertiary teaching hospital. Multicentric future studies are encouraged to compare between different sectors, including the private sector, governmental hospitals and military hospitals. Moreover, we did not include patients undergoing emergency surgeries, as this patient group requires separate strict inclusion criteria and a different study design. The relationship between the COVID-19 pandemic and preoperative anxiety is continuously changing, for which the results of our study might be limited to the epidemiological status of the COVID-19 pandemic in the setting in which the study was conducted. This investigation did not include transient psychophysiological disorders, including transient signs and symptoms, as well as changes in patients’ vital signs during the preoperative period. Future studies investigating these variables are encouraged to take into consideration patients’ medical illnesses, their current medications, and preoperative premedication.

## 5. Conclusions

In conclusion, this study demonstrated that 30.1% of patients have high preoperative anxiety, with a rate of 37.4% among females, compared to 19% among males. Fear of pain after surgery was the most common factor related to anxiety on the day of surgery. This suggests that thorough preoperative counseling about post-operative pain management strategies can play a crucial role in decreasing preoperative anxiety. The rate of vaccination against COVID-19 among our adult operative population at the time of this investigation was 82.2%. Although the current study suggests that there is no significant correlation between APAIS-A-T score and previous COVID-19 infection or vaccination, being afraid of contracting COVID-19 during hospital stay was positively associated with high preoperative anxiety, for which controlling the spread of COVID-19 by implementing all preventive measures can also play a role in decreasing preoperative anxiety during this pandemic.

## Figures and Tables

**Table 1:** A comparison between patients who had high preoperative anxiety and low preoperative anxiety in the demographic data.

Characteristics		Anxiety Level *		Total (*n* = 794)	*p* Value
		Low Anxiety (*n* = 555)	High Anxiety (*n* = 239)		
Demographic data					
Age (years)		46.5 ± 16.1	41.2 ± 15.4	44.9 ± 16.1	<0.001
Gender	Male	255 (45.9)	60 (25.1)	315 (39.7)	<0.001
	Female	300 (54.1)	179 (74.9)	479 (60.3)	
Marital status	Married	440 (79.3)	170 (71.1)	610 (76.8)	0.079
	Single	93 (16.8)	55 (23.0)	148 (18.6)	
	Widow	15 (2.7)	8 (3.3)	23 (2.9)	
	Divorced	7 (1.3)	6 (2.5)	13 (1.6)	
Educational level	Illiterate	11 (2.0)	1 (0.4)	12 (1.5)	0.134
	Primary school	41 (7.4)	10 (4.2)	51 (6.4)	
	Secondary school	174 (31.4)	70 (29.3)	244 (30.7)	
	Middle school	48 (8.6)	21 (8.8)	69 (8.7)	
	Bachelors/college or higher	281 (50.6)	137 (57.3)	418 (52.6)	
Employment status	Unemployed	267 (48.1)	117 (49.0)	384 (48.4)	0.827
	Employed	288 (51.9)	122 (51.0)	410 (51.6)	
Occupation field (*n* = 410)	Non-medical field	262 (91.0)	105 (86.1)	367 (89.5)	0.138
	Medical field	26 (9.0)	17 (13.9)	43 (10.5)	
Residency	Capital city	331 (59.6)	144 (60.3)	475 (59.8)	0.872
	Governorates	224 (40.4)	95 (39.7)	319 (40.2)	
Monthly income	Less than 500 JOD	288 (51.9)	125 (52.3)	413 (52.0)	0.903
	500–1000 JOD	215 (38,7)	94 (39.3)	309 (38.9)	
	More than 1000 JOD	52 (9.4)	20 (8.4)	72 (9.1)	
Anesthetic and surgical history					
Type of upcoming surgery	Minor surgical procedure	309 (55.7)	123 (51.5)	432 (54.4)	0.274
	Major surgical procedure	246 (44.3)	116 (48.5)	362 (45.6)	
Type of upcoming anesthesia	General	468 (84.3)	208 (87.0)	676 (85.1)	0.326
	Regional/neuraxial	87 (15.7)	31 (13.0)	118 (14.9)	
Surgical discipline	Cardiac and vascular surgery	21 (3.8)	12 (5.0)	33 (4.2)	0.056
	Endocrine surgery	11 (2.0)	6 (2.5)	17 (2.1)	
	Gastrointestinal surgery	92 (16.6)	45 (18.8)	137 (17.3)	
	Neurological surgery	38 (6.8)	15 (6.3)	53 (6.7)	
	Gynecological and obstetric surgeries	108 (19.5)	58 (24.3)	166 (20.9)	
	Oncological surgery	5 (0.9)	5 (2.1)	10 (1.3)	
	Orthopedics surgery	79 (14.2)	37 (15.5)	116 (14.6)	
	Otolaryngology surgery	51 (9.2)	25 (10.5)	76 (9.6)	
	Plastic surgery	30 (5.4)	10 (4.2)	40 (5.0)	
	Urological surgery	120 (21.6)	26 (10.9)	146 (18.4)	
Previous history of surgery under anesthesia		443 (79.8)	169 (70.7)	612 (77.1)	0.005
Number of previous surgeries (*n* = 612)	One	153 (34.5)	66 (39.1)	219 (35.8)	0.191
	Two	105 (23.7)	46 (27.2)	151 (24.7)	
	Three or more	185 (41.8)	57 (33.7)	242 (39.5)	
Previous history of surgical complications (*n* = 612)		31 (7.0)	17 (10.1)	48 (7.8)	0.208
Previous history of anesthetic complications (*n* = 612)		18 (4.1)	13 (7.7)	31 (5.1)	0.067
Family members or friends ever experienced any surgical complications		28 (5.0)	19 (7.9)	47 (5.9)	0.112
Family members or friends ever experienced any anesthetic complications		16 (2.9)	12 (5.0)	28 (3.5)	0.134
APAIS-A-T		6.0 ± 2.2	14.3 ± 2.7	8.5 ± 4.5	<0.001
APAIS-A-An		2.7 ± 1.2	6.6 ± 2.7	3.9 ± 2.5	<0.001
APAIS-A-Su		3.3 ± 1.8	7.7 ± 2.2	4.6 ± 2.8	<0.001
APAIS-I		4.5 ± 2.8	7.0 ± 2.8	5.3 ± 3.0	<0.001

* Patients with Amsterdam preoperative anxiety and information scale about anesthesia and surgery total score <10 were considered as the low preoperative anxiety group, while patients with APAIS-A-T ≥11 were considered as the high preoperative anxiety group. Numbers are presented as number (percent) and mean ± standard deviation. JOD: Jordanian dinars; PAIS-A-T: Amsterdam preoperative anxiety and information scale about anesthesia and surgery total score; PAIS-A-An: APAIS anxiety about anesthesia score; APAIS-A-Su: APAIS anxiety about surgery score; APAIS-I: APAIS information score.

**Table 2:** A comparison between different surgical specialties in Amsterdam preoperative anxiety and information scale about anesthesia and surgery.

Surgical Characteristics	Number (Percent)	APAIS-A-T	APAIS-A-An	APAIS-A-Su	APAIS-I
Procedure type					
Minor surgical procedure	432 (54.4)	8.3 ± 4.4	3.8 ± 2.5	4.5 ± 2.7	5.2 ± 3
Major surgical procedure	362 (45.6)	8.8 ± 4.6	4 ± 2.5	4.8 ± 2.9	5.4 ± 3
*p* value	0.274	0.125	0.364	0.213	0.35
Surgical specialty					
Cardiac and vascular surgery	33 (4.2)	9.2 ± 4.9	4.6 ± 2.9	4.6 ± 2.6	5.3 ± 3.2
Orthopedics surgery	116 (14.6)	8.7 ± 4.7	3.9 ± 2.6	4.8 ± 3.1	5.1 ± 3
Urological surgery	146 (18.4)	7.1 ± 3.9	3.4 ± 2.2	3.7 ± 2.4	4.9 ± 3.1
Plastic surgery	40 (5.0)	7.7 ± 4.3	3.2 ± 2.1	4.5 ± 3.2	5 ± 3.2
Gastrointestinal surgery	137 (17.3)	8.8 ± 4.3	4 ± 2.4	4.7 ± 2.6	5.6 ± 2.8
Otolaryngology surgery	76 (9.6)	8.4 ± 4.1	4.1 ± 2.6	4.3 ± 2.7	5.3 ± 2.9
Gynecological and obstetric surgeries	166 (20.9)	9.4 ± 4.7	4.1 ± 2.6	5.3 ± 2.9	5.5 ± 3.1
Neurosurgery	53 (6.7)	8.7 ± 5	3.7 ± 2.7	5 ± 3	4.5 ± 2.8
Endocrine surgery	17 (2.1)	8.8 ± 3.5	3.7 ± 1.7	5.1 ± 2.6	6.3 ± 3.2
Oncological surgery	10 (1.3)	11.5 ± 5.2	5.6 ± 3.6	5.9 ± 3.1	6.2 ± 3.3
*p* value	0.056	<0.001	0.02	<0.001	0.139

PAIS-A-T: Amsterdam preoperative anxiety and information scale about anesthesia and surgery total score; PAIS-A-An: APAIS anxiety about anesthesia score; APAIS-A-Su: APAIS anxiety about surgery score; APAIS-I: APAIS information score. Numbers are presented as mean ± standard deviation.

**Table 3:** Factors associated with preoperative anxiety.

Characteristics	Anxiety Level *		Total (*n* = 794)	*p* Value
	Low Anxiety (*n* = 555)	High Anxiety (*n* = 239)		
Factors making them anxious on surgery day				
Fear of surgical complication	123 (22.2)	129 (54.0)	252 (31.7)	<0.001
Fear of anesthesia	82 (14.8)	119 (49.8)	201 (25.3)	<0.001
Fear of becoming infected with COVID-19	50 (9.0)	47 (19.7)	97 (12.2)	<0.001
Fear of doctors’ mistake	115 (20.7)	106 (44.4)	221 (27.8)	<0.001
Fear of pain after surgery	156 (28.1)	118 (49.4)	274 (34.5)	<0.001
Fear of death	28 (5.0)	64 (26.8)	92 (11.6)	<0.001
None of the above	241 (43.4)	22 (9.2)	263 (33.1)	<0.001
Factor that makes them the most anxious				
Fear of anesthesia	56 (10.1)	81 (33.9)	137 (17.3)	<0.001
Fear of surgical complication	115 (20.7)	103 (43.1)	218 (27.5)	
Fear of becoming infected with COVID-19	22 (4.0)	7 (2.9)	29 (3.7)	
None of the above	362 (65.2)	48 (20.1)	410 (51.6)	

* Patients with Amsterdam preoperative anxiety and information scale about anesthesia and surgery total score <10 were considered as the low preoperative anxiety group, while patients with APAIS-A-T ≥11 were considered as the high preoperative anxiety group. Values are presented as number (percent). COVID-19: coronavirus disease 2019.

**Table 4:** History of COVID-19 and COVID-19 vaccination and their relationship with preoperative anxiety.

Characteristics		Anxiety Level *		Total (*n* = 794)	*p* Value
		Low Anxiety (*n* = 555)	High Anxiety (*n* = 239)		
Previous history of COVID-19		154 (27.7)	75 (31.4)	229 (28.8)	0.3
Symptoms experienced (*n* = 229)	No symptoms	17 (11.0)	8 (10.7)	25 (10.9)	0.45
	Mild (flu-like symptoms: fever, fatigue, myalgia, cough, runny nose)	93 (60.4)	39 (52.0)	132 (57.6)	
	Moderate (persistent fever, productive cough, wheezing)	38 (24.7)	26 (34.7)	64 (27.9)	
	Severe (hospitalization was needed for ICU and respiratory support)	6 (3.9)	2 (2.7)	8 (3.5)	
Previous complication of COVID-19 (*n* = 229)		17 (11.0)	11 (14.7)	28 (12.2)	0.432
Family members or friends experienced previous complication of COVID-19		133 (24.0)	83 (34.7)	216 (27.2)	0.002
Vaccinated against COVID-19		466 (84.0)	187 (78.2)	653 (82.2)	0.053
Fear of becoming infected with COVID-19 during their stay in the hospital		103 (18.6)	64 (26.8)	167 (21.0)	0.009
Fear of becoming infected with COVID-19 made them hesitate having the surgery		17 (3.1)	19 (7.9)	36 (4.5)	0.002
Having a vaccine against COVID-19 made them feel safer about having surgery		236 (50.3)	107 (56.0)	343 (52.0)	0.184

* Patients with Amsterdam preoperative anxiety and information scale about anesthesia and surgery total score <10 were considered as the low preoperative anxiety group, while patients with APAIS-A-T ≥11 were considered as the high preoperative anxiety group. Values are presented as number (percent). COVID-19: coronavirus disease 2019.

**Table 5:** Univariable and multivariable regression analysis for factors correlating with high preoperative anxiety.

Variable	Univariable Regression				Univariable Regression			
	OR	95% CI for OR		*p* Value	OR	95% CI for OR		*p* Value
		Lower	Upper			Lower	Upper	
Age	0.98	0.97	0.99	<0.001	0.98	0.97	0.99	<0.001
Gender (Male)	0.39	0.28	0.55	<0.001	0.39	0.28	0.55	<0.001
Educational level *				0.169	-	-	-	-
Educational level (secondary school)	4.43	0.56	34.92	0.158	-	-	-	-
Educational level (bachelor’s/college or higher)	5.36	0.69	41.96	0.110	-	-	-	-
Educational level (middle school)	4.81	0.58	39.71	0.144	-	-	-	-
Educational level (primary school)	2.68	0.31	23.28	0.371	-	-	-	-
Previous history of surgery under anesthesia	0.61	0.43	0.86	0.005	0.63	0.43	0.91	0.014
Previous history of surgical complications	1.49	0.80	2.76	0.210	-	-	-	-
Previous history of anesthetic complications	1.97	0.94	4.11	0.072	-	-	-	-
Previous history of COVID-19	1.19	0.86	1.66	0.300	-	-	-	-
Vaccinated against COVID-19	0.69	0.47	1.01	0.054	-	-	-	-
Fear of becoming infected with COVID-19 during their stay in the hospital	1.60	1.12	2.29	0.009	1.47	1.02	2.14	0.041

OR: odds ratio; 95% CI: 95% confidence interval; COVID-19: coronavirus disease 2019. *: We used the educational level “Illiterate” as the reference standard for all comparisons.

## Data Availability

The data presented in this study are available on request from the corresponding author.

## Supplementary Materials

The following supporting information can be downloaded at: https://www.mdpi.com/article/10.3390/healthcare10030515/s1, Table S1: Amsterdam Preoperative Anxiety and Information Scale (APAIS).
