# Supplementary material for: Preoperative Anxiety among Adult Patients Undergoing Elective Surgeries at a Tertiary Teaching Hospital: A Cross-Sectional Study during the Era of COVID-19 Vaccination
Source: Healthcare (Basel). 2022 Mar 11;10(3):515. doi: 10.3390/healthcare10030515 (PMC8950895; doi:10.3390/healthcare10030515)
Supplement: Supplementary file 1 [file healthcare-10-00515-s001.zip › healthcare-1606651-supplementary.pdf]

healthcare MDPI

Table S1. Amsterdam Perioperative Anxiety and Information Scale (APAIS).

The Amsterdam Preoperative Anxiety and Information Scale	Not at All	1	2	3	4	5	Very Much
1 - I am worried about the anesthesia.							
2 - The anesthesia is on my mind continually.							
3 - I would to like to know as much as possible about the Anastasia.							
4 - I am worried about the procedure.							
5 - The procedure is on my mind continually.							
6 - I would like to know as much as possible about the procedure.							
